# Carbon monoxide intoxication with a CO-Hb of 30% while smoking waterpipe: a case report

**DOI:** 10.1186/s12245-023-00560-7

**Published:** 2023-11-07

**Authors:** Ghaith Mohsen, Michael Kemmerer, Lars Eichhorn

**Affiliations:** 1 Department of Anaesthesiology and Intensive Care Medicine, University Hospital Bonn, Bonn, Germany; 2 Druckkammerzentrum Rhein Main Taunus GmbH, Wiesbaden, Germany; 3 Department of Anaesthesiology and Intensive Care Medicine, Helios Hospital Bonn/Rhein-Sieg, Bonn, Germany

**Keywords:** Carbon monoxide, CO, Hyperbaric therapy, HBOT, Shisha, Waterpipe

## Abstract

Carbon monoxide (CO) poisoning is a significant public health issue and a considerable economic burden in developed countries. While the majority of non-fire-related CO poisonings are attributed to gas heating, there are several other less recognized sources that should be considered in the initial differential diagnosis.

The patient in this case was a 21-year-old who experienced a brief episode of loss of consciousness and was subsequently admitted to the Emergency department. Upon evaluation, the patient was diagnosed with CO poisoning, which necessitated hyperbaric oxygen therapy to mitigate the effects of this toxic exposure.

Despite exhibiting harmful symptoms initially, the patient stated in a phone interview two and a half years post-incident that they have not experienced any enduring effects such as cardiac arrhythmia or concentration deficits. While their understanding of the risks associated with waterpipe smoking has increased, it has not influenced any major changes in their waterpipe smoking habits.

## Introduction

Carbon monoxide is an odorless and colorless gas with a molecular weight similar to that of air. Although CO poisoning is usually caused by fires and defective heating systems, poisoning due to smoking water pipes has been increasingly seen in recent years.

There are ca. 50,000 documented CO Poisonings annually in the USA, the worldwide cumulative incidence and mortality of CO poisoning are estimated at 137 and 4.6 deaths per million, respectively [1].

When inhaled, CO Is diffused through the alveolar membrane and binds to hemoglobin with an affinity that is 200–300 higher than that of oxygen. CO then obstructs oxygen transport and delivery [2]. The acute effects of CO poisoning are thus due to tissue hypoxia [3]. The symptoms of CO Poisoning most commonly include headache, dizziness, fatigue, nausea, chest pain, dyspnoea, and loss of consciousness [4].

Waterpipe smoking has become more common in recent years, although there are many cases reporting CO poisoning from hookah smoking, it is still frequently overseen as a risk factor.

Carbon monoxide poisoning is responsible for a significant health and economic burden in Europe. in 2021, CO was the cause of death for 397 people in Germany [5]. According to the CDC (Centre of Disease Control), around 430 people die of CO poisoning every year with nearly 50,000 emergency department visits due to CO poisoning [6–8].

Although the majority of non-fire related poisonings are due to Gas heating in homes. Lesser common sources should be included in the initial differential diagnosis when patients present with similar symptoms and signs of CO exposure.

Hookah and water pipe smoking has become more common among young adults in Western Europe. With a lifetime prevalence of 63% in adults in Germany between 18 and 25 years of age [9].

Smoking tobacco through a waterpipe can lead to significantly higher levels of harmful chemicals compared to smoking a cigarette. Research indicates that smoking a waterpipe for an hour can expose the individual to around 145 mg of carbon monoxide, which is nearly eight times the concentration found in a single cigarette. Moreover, waterpipe smoke contains higher levels of other toxic substances such as nicotine, tar, chrysene, phenanthrene, and fluoranthene [10]. The primary reason for the high levels of ambient CO generated by water pipe smoking, surpassing those of other combustible smoking methods, is due to the use of charcoal as the source of heat [11].

In this article, we report a case of a patient who suffered from severe carbon monoxide poisoning causing a collapse at home while smoking a water pipe. The patient’s carboxyhemoglobin (COHb) level was measured at 30.4% in arterial blood gas, 2 h after the incident. The patient also experienced a hypertensive crisis, tachycardia, and frequent ventricular extrasystoles, which were observed through preclinical and clinical electrocardiograms (ECGs). To treat the patient’s condition, hyperbaric oxygen therapy was administered over three sessions, which resulted in a successful recovery.

## Case report

A 21-year-old male with no past medical history and no regular medications was brought to the emergency department via ambulance after experiencing a 10 second episode of unconsciousness at home, following the smoking of a waterpipe containing tobacco. Prior to collapsing, the patient reported a sudden onset of dizziness and mild headaches. The patient denied experiencing angina pectoris, dyspnea, or palpitations. The family history was positive for coronary artery disease on the father’s side. Upon their arrival, the ambulance team documented the patient’s vital signs, including a temperature of 36.0 °C, heart rate of 125/min, respiratory rate of 18/min, blood pressure of 180/130 mmHg, and a pulse oximetry reading of 94% while breathing room air. As far as we know, the carbon monoxide (CO) levels in the patient’s home were not dangerously high, as they did not trigger the alarm of the CO detector carried by the ambulance team set to 60 ppm.

Upon evaluation, the patient was found to be conscious and fully aware, scoring 15 on the Glasgow coma scale. No physical injuries were observed as a result of the fall.

An electrocardiogram (ECG) showed sinus tachycardia with 125/min with frequent ventricular extrasystoles (VES), normal axis, normal P, PQ, ORS-segments, and also normal ST/T segments and QT time. The APACHE II Score added up to a 6 and the poison severity score on admission added up to 3.

The patient reported a history of tobacco use, consuming one pack daily over the past 3 years. Additionally, he smokes a water pipe approximately three times a week. He also disclosed the consumption of 2–3 units of alcohol on 1–2 occasions weekly. The use of recreational drugs was denied.

Furthermore, the patient’s complete blood count, serum chemistry panel, and cardiac enzyme levels were all within normal limits and showed no abnormalities. The results of the urine drug panel were also unremarkable.

**Fig. 1:**
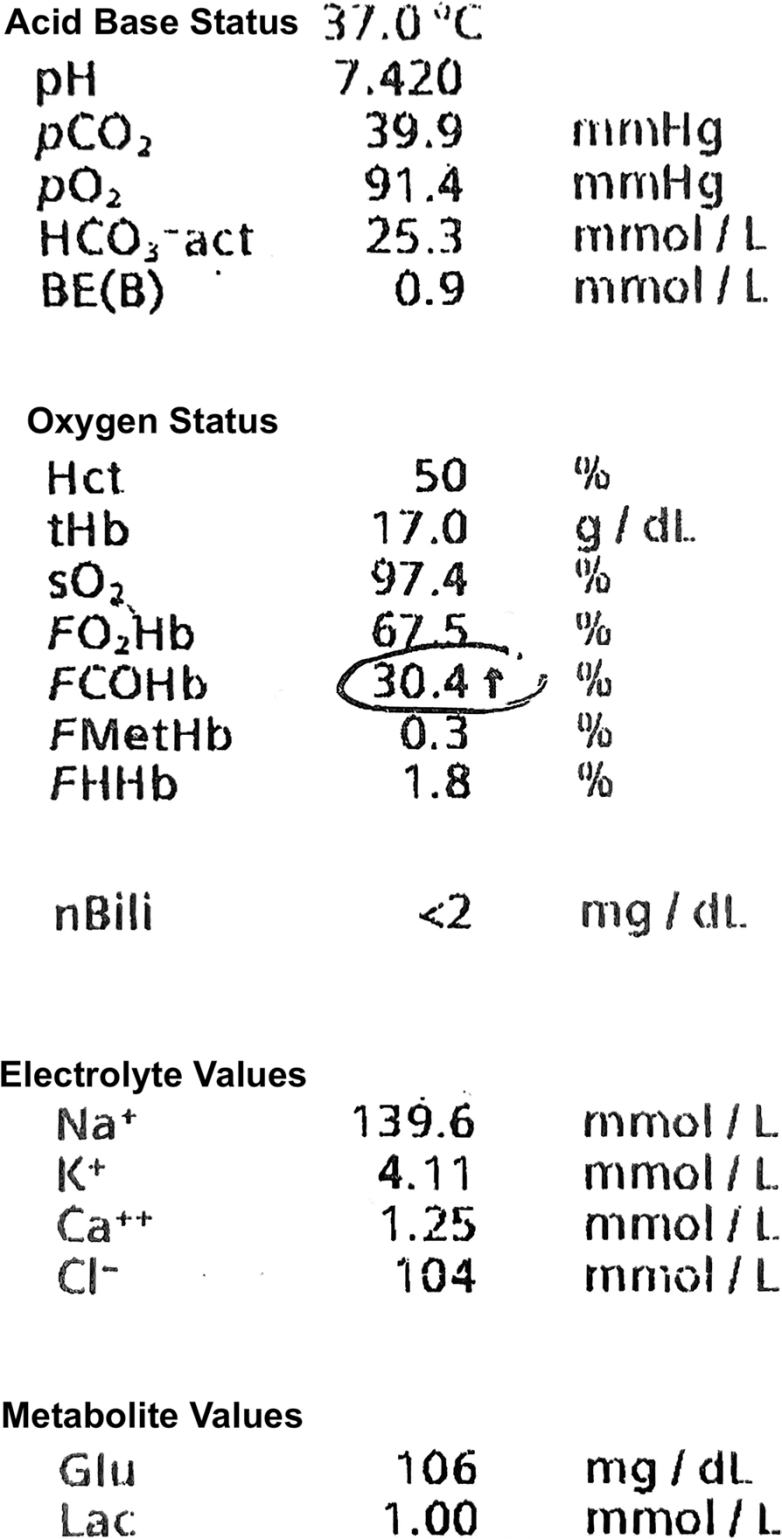
Arterial blood gas showing a CO-Hb of 30.4%

The first arterial blood gas (ABG) (Fig. 1.) was obtained 2 h after arrival in the emergency department, which revealed a COHb of 30.4%, pH 7.42, pCO2 39.9 mmHg, pO2 91.4 mmHg, and a Lactate of 1.0 mmol/l. As a result, the patient was promptly started on oxygen therapy using a simple face mask and was then transported to the next hyperbaric oxygen therapy (HBO) center.

Upon admission to the hyperbaric center, the COHb was measured to be 17.2%. The HBO therapy was initiated nearly 4 h after the patient’s initial admission to the ED, by which time the patient's headaches had already subsided.

The patient underwent three hyperbaric oxygen therapy sessions, each lasting for 145 min at 2.4 atmospheres with 100% FiO2. Following the completion of the therapy, the patient was released and exhibited no symptoms or complaints. His vital signs before discharge were unremarkable.

Two and a half years after the incident, the patient participated in an interview to discuss his experience. He reported that the symptoms initially manifested as a brief episode of dizziness and headache, followed by a rapid loss of consciousness. Upon receiving oxygen, the patient experienced a slight improvement in his condition. Subsequent hyperbaric oxygen therapy (HBOT) led to a marked recovery, with the patient becoming symptom-free after completion of the treatment course. Normal pulse rate was restored after the entire therapy was concluded.

The patient continues to engage in water pipe smoking, albeit less frequently and exclusively outdoors. The incident did not result in any alterations in his behavior or that of his friends and acquaintances. Furthermore, the patient reported no noticeable long-term health complications, such as memory impairment, heart palpitations, or an increase in the frequency of illness or common cold, following the incident.

## Discussion

Carbon monoxide poisoning is known as the most common fatal inhaled intoxicating gas with no available antidote. The severity of poisoning depends on the length of the exposure period and its concentration in the air [12]. The main effect is displacing oxygen in the haemoglobin and the generation of Carboxyhaemoglobin, which blocks oxygen from being delivered to the tissues leading to hypoxia [13, 14]. Fatality rates increase with age and reach their highest in people older than 65 years. Those who are suffering from cardiac and respiratory diseases could be more susceptible to a worse outcome. Especially patients with COPD who have a ventilation-perfusion abnormality and may develop a respiratory depressive response to 100% oxygen therapy [13]. Cardiac effects could arise from CO Poisoning as well; as up to one-third of patients with moderate to severe CO poisoning show myocardial injury. [13, 15, 16].

In a study by Satran et al., 30 of 53 patients with moderate to severe CO poisoning had abnormal left ventricular function [17].

The current treatment approach for CO poisoning involves administering 100% normobaric oxygen (NBO2) or hyperbaric oxygen (HBO2) at 2.5–3 atmospheres of pressure [14]. The German national guidelines for CO-poisoning therapy recommend the initiation of therapy within 6 h of the initial diagnosis, suggesting a regimen of three therapy sessions within a 24-h period [18]. NBO2 therapy can be delivered via a non-rebreather mask or endotracheal intubation. The idea of this therapy is to remove CO from Hb as quickly as possible thereby reducing the risk of tissue hypoxia caused by oxygen deprivation. NBO2 and HBO2 remove CO at a faster pace by augmenting the partial pressure of inhaled oxygen which in turn increases the dissociation of CO from Hb [4, 14]. HBO2 has shown a reversal of inflammation and dysfunction of the mitochondria induced by CO [19, 20].

A study by Weaver et al. [21] showed a remarkable improvement in neurocognitive dysfunction caused by CO in patients who went through HBO in comparison to those who did not.

Rose et al. proposed an innovative therapeutic strategy utilizing a specific antidote, Ngb-H64Q-CCC, which is a recombinant neuroglobin. This modified neuroglobin exhibits an affinity for carbon monoxide (CO) that is approximately 500 times greater than that of haemoglobin. The study demonstrated that Ngb-H64Q-CCC could effectively scavenge CO from both molecular haemoglobin and haemoglobin encapsulated within packed red blood cells. This intervention effectively mitigated the inhibitory effects of CO on mitochondrial respiration. Subsequently, the CO-bound form of the antidote undergoes renal excretion. Experimental trials conducted on rodents yielded promising outcomes, indicating the potential efficacy of this approach [22].

Although waterpipe smoking remains a relatively unknown source of CO poisoning, several studies demonstrated its rising incidence.

A single-center retrospective cohort study by Eichhorn et al. found a significant number of CO poisoning cases related to waterpipe smoking, indicating that this source of CO poisoning should be considered in the emergency department, especially in young patients presenting with non-specific neurological symptoms [23].

This was also demonstrated in a retrospective analysis at a New York City HBO center, which found a significant increase in waterpipe-related carbon monoxide poisoning among young, otherwise healthy individuals, with waterpipe smoking becoming the leading cause of CO poisoning [24]. The increase in waterpipe-associated CO cases may be due to the rising popularity of hookah establishments [24].

These studies suggest that waterpipe-associated CO poisoning might very well be more common in industrial countries than accredited.

This case report possesses a few limitations. A notable one is the utilization of a telephone interview for patient assessment, rather than employing standardized neuropsychologic tests. An in-person examination was precluded due to the associated inconvenience of long-distance travel.

In the case above, the young patient exhibited classic symptoms of acute CO poisoning and had smoked water pipe, yet the COHb testing was performed 2 h after he arrived in the emergency department, ultimately delaying the initiation of HBO therapy. Nevertheless. The HBO therapy began 4 h after the initial admission, adhering to the recommended timeframe by the German guidelines for therapy of CO poisoning.

With this growing trend of waterpipes, it is crucial for paramedics and emergency care staff to be well-informed to identify and manage such cases. In situations where water pipe-related CO poisoning is suspected, initiating oxygen therapy should be considered a priority until the diagnosis can be either confirmed or ruled out.

This reported case highlights a delayed diagnosis of CO poisoning resulting from insufficient knowledge and recognition of the possibility of CO poisoning originating from a source that, despite its apparent increase, continues to be predominantly unrecognized.

To our knowledge, there are no documented deaths due to CO poisoning by water pipe smoking, but it still presents an important and easily overseen cause of CO poisoning.

## Data Availability

All data are available as part of the article and no additional data are required.
